# In Situ Anchored, Ultrasmall, Oxygen Vacancy-Rich TiO_2−x_ on Carbonized Bacterial Cellulose for the Efficient Adsorption and Separation of Organic Pollutants

**DOI:** 10.3390/nano15070514

**Published:** 2025-03-28

**Authors:** Man Zhou, Yanli Zhou, Minmin Ni, Yuzhe Zhang, Song Xu, Hao Ma, Jian Zhou, Jin Zhao, Liwei Lin, Zhongyu Li

**Affiliations:** 1 Jiangsu Province Key Laboratory of Fine Petrochemical Engineering, Changzhou University, Changzhou 213164, China; s22020817024@smail.cczu.edu.cn (Y.Z.); s22020860046@smail.cczu.edu.cn (M.N.); yuzhez@cczu.edu.cn (Y.Z.); xusong@cczu.edu.cn (S.X.); lin-official@cczu.edu.cn (L.L.); 2 BGRIMM Technology Group, Daxing, Beijing 102600, China; haoma@bgrimm.com; 3 Zhongxi Rare Earth New Materials Co., Ltd., Changzhou 213164, China; xxt2025@outlook.com; 4 School of Materials Science and Engineering, Nanjing University of Posts and Telecommunications, Nanjing 210023, China; iamjzhao@njupt.edu.cn

**Keywords:** carbonized bacterial cellulose, oxygen vacancies, high selectivity, dye adsorption/separation

## Abstract

Superior selective adsorption of organic dye is still a big challenge in the process of dye wastewater treatment. Meanwhile, low-price and environmentally friendly biomass-based adsorbents show huge potential in the fields of separation and purification. In this study, we adopted the “hydrolysis–calcination method” to develop a novel in situ anchoring strategy for ultrasmall TiO_2−x_ on carbonized bacterial cellulose (CBC), which was derived from natural bacterial cellulose. Notably, 3D networks of porous CBC played a dual role for both providing hydrolytic sites and controlling the oxygen vacancies (V*_o_*) of TiO_2−x_. As for the single-dye adsorption, the TiO_2−x_/CBC had a strong adsorption ability (101.4 mg/g) for removing methylene blue (MB), which was much higher than that of methyl orange (MO), malachite green (MG), rhodamine B (RhB), and tetracyclines (TC). Moreover, under the optimized carbonization temperature (T*_c_*) of 300 °C, the TiO_2−x_/CBC-300 exhibited an outstanding separation efficiency of 97.07% for the MB/MO solution. Detailed analysis confirmed that T*_c_* was a key regulator for adjusting the V*_o_* concentration, which directly influenced the surface charge density and, further, the separation efficiency of TiO_2−x_/CBC. Additionally, the used adsorbent could be easily regenerated from washing by ethanol. After 4 regenerations, the adsorption efficiency declined only by 6.9% after 20 min and 13.6% after 120 min adsorption, respectively. Ultimately, this oxygen vacancy-rich TiO_2−x_/BC system illuminated good prospects for mixed dye wastewater adsorption and separation.

## 1. Introduction

On a global scale, the textile, printing, and dyeing industry still discharges the majority of industrial wastewater [1]. In industrial production, such as printing and dyeing and textile manufacturing, organic dyes such as methyl orange (MO), malachite green (MG), and rhodamine B (RhB) are widely used as raw materials. The wastewater containing these dyes, which is discharged after production, seriously threatens the natural environment and the health of all living organisms [2]. Wastewater from printing and dyeing is characterized by its large volume, complex composition, as well as high chromaticity, which increases the difficulty of water purification [3]. According to the acceptable limits of many dyes (0.01–0.05 mg/L) [4] for industrial discharge, some new treatment processes were widely reported alongside conventional methods, such as photocatalysis methods, biosorption methods [5], electrochemical methods, and electron beam treatments [6]. However, some disadvantages (e.g., high costs, complex operation, and low selectivity) limit the further application of these wastewater treatment technologies, especially for particular purposes like rapidness, simplicity, reusability, and high selectivity during the purification process [7].

In recent years, physical adsorption methods have been deeply studied, with the development of various inorganic adsorbents for dye wastewater treatment [8]. As a class of typical adsorbents, porous carbon materials derived from natural biomass become an efficient solution in the field of adsorption due to their abundant pore structures, good stability, and reusability [9]. For example, porous N-doped carbons (NPCs) derived from the bottlebrush flower reached a large specific surface area (~2025 m^2^·g^−1^), which exhibited high efficiency in removing binary organic pollutants [10]. Similarly, natural biomass-derived, carbon-based adsorbents have been studied extensively, such as eggplant-based porous carbon materials (~560 m^2^·g^−1^) [11], coconut-based carbon materials (~900 m^2^·g^−1^) [12], bamboo-based carbon adsorbents (~933 m^2^·g^−1^) [13], corncob-based carbon adsorbents (~1722 m^2^·g^−1^), etc. [14]. Different from these natural plant, bacterial cellulose (BC), with its unique 3D cross-linked structure, is an ideal candidate for dye wastewater treatment [15]. The key advantage of BC is its special synthesis process of bacterial fermentation, which leads to a high purity and high hydroxyl density. Based on the methods of chemical modification, hydroxyl groups play an important role for the in situ growth of metal hydroxide and metallic oxide [16]. In the aqueous solution system, MB ionizes to release a cation (-N^+^(CH_3_)_3_), while MO exists in an anionic state due to the ionization of the sulfonate group (-SO_3_⁻). The OH groups on the surface of the nanomaterial can enhance the adsorption of MB cations. For example, several BC-derived composites, such as poly(vinyl amine)-grafted BC (PVAm-g-BC) [17], achieve a 96.5% MB adsorption efficiency. FeOOH/carbonized bacterial cellulose (FeOOH/CBC) [18] shows a 96.2% MO adsorption efficiency. These materials efficiently remove single-dye components. However, these physical adsorption processes can hardly achieve selective dye adsorption due to their lack of functional adsorption sites [19].

In practice, real textile and dye wastewaters always present as a mixture, containing different kinds of cationic, anionic, and neutral dyes [20]. Compared with non-selective adsorption, a highly selective adsorption process with a simple and effective regeneration ability is one of the ideal solutions but is still a challenging task. Till now, some adsorbents with selective adsorption properties have been reported by anchoring semiconductors as functional sites, such as MIL-101(Fe) [21], La_2_Mo_2_O_9_ [22], ZIF-67 [23], carbon microtubes [24], etc. However, these artificial nanostructures still have some hindering factors, including their high cost, high toxicity, and complex synthesis, which prevent their industrial applications. Different from the nanostructures mentioned above, TiO_2_ is an old but newly developed semiconductor covering many fields [25], such as heterogeneous catalysis [26], photocatalysis [27], electrocatalysis [28], etc. Over the past decades, TiO_2_ semiconductors, especially nano-TiO_2_, have been used as traditional adsorbents [29]; this combination provided two obvious advantages. Firstly, both TiO_2_ and carbon materials are eco-friendly materials. Secondly, owing to the low densities of both two components, TiO_2_-anchored carbon composites have great potential for the highly efficient adsorption and separation process [30,31]. For over a decade, TiO_2_-based materials were designed and constructed with unique morphologies (e.g., hollow microspheres [32], mesoporous structures [33,34], and nanotubes [35]). To overcome the shortcomings of low selective adsorption on the surface of pure TiO_2_, researchers focused on surface and interface chemistry, including amino modification [36], co-doping [37,38], Ag_2_O modification [39], bimetallic alloy deposition [40], etc. Overall, TiO_2_-based adsorbents with a low price, high selectivity, and easy regeneration have rarely been reported.

Herein, a new class of 3D carbonized bacterial cellulose (CBC)-supported TiO_2_-based composites (TiO_2−x_/CBC) was synthesized using the “hydrolysis–calcination method”. Natural BC fibers with a large surface area and rich hydroxyl groups could provide ideal anchoring sites during the in situ hydrolysis process. Benefiting from the highly dispersed precursor, the carbonization temperature (T*_c_*) acted as a key regulator for adjusting both the degree of carbonization and the concentration of oxygen vacancy. In the field of materials science, chemical vacancies (acidic sites) and electronic vacancies (tuning d-band center) have a significant impact on the characteristics of oxygen vacancies. As acidic sites, chemical vacancies can form bonds with oxygen atoms by providing protons or electrons, facilitating the desorption of oxygen atoms and, thus, contributing to the formation of oxygen vacancies. Electronic vacancies, on the other hand, can influence the interaction between oxygen vacancies and surrounding atoms by adjusting the energy center of the d-band, thereby altering the catalytic performance related to oxygen vacancies. The relationship between the state of surface charge and the adsorption performance of TiO_2−x_/CBC was evaluated under four different conditions of T*_c_* from 200 °C to 500 °C. The optimized TiO_2−x_/CBC adsorbent showed excellent selective performance and good cycle ability for separating the binary mixtures of MB/MO.

## 2. Materials and Methods

### 2.1. Materials

All chemicals utilized in the experiment were of analytical-grade purity and required no further purification. Tetrabutyl titanate (TBT, C_16_H_36_O_4_Ti), anhydrous ethanol (C_2_H_6_O), anhydrous methanol (CH_4_O), hydrochloric acid (HCl), sodium hydroxide (NaOH), methylene blue (MB, C_16_H_18_ClN_3_S), methyl orange (MO, C_14_H_14_N_3_NaO_3_S), malachite green (MG, C_23_H_25_ClN_2_), rhodamine B (RhB, C_28_H_31_ClN_2_O_3_), and tetracycline (TC, C_22_H_24_N_2_O_8_) were procured from Shanghai Aladdin Biochemical Technology Co., Ltd. (Shanghai, China). Biomass from BC membranes was sourced from Guilin Qihong Co., Ltd. (Guilin, China). Liquid nitrogen was purchased from Changzhou Huayang Gas Co., Ltd. (Changzhou, China). Distilled water was used throughout the experimentation. The freeze dryer (FD-2A) was purchased from Shanghai Bilang Instrument Manufacturing Co., Ltd. (Shanghai, China), and the tube furnace (GSL-1100X-S) was purchased from Hefei Kejing Materials Technology Co., Ltd. (Heifei, China)

### 2.2. Purification of BC

To remove residual impurities from the fibers of the purchased BC, a purification process was followed before the growth of TiO_2_. Initially, an NaOH solution with a concentration of 2% was prepared. Then, the BC membranes were immersed in the NaOH solution at 90 °C for 1 h. After cooling down to room temperature, the BC membranes were carefully washed with deionized water until it reached a neutral pH [41]. To maintain the 3D structure of the biomass, the purified BC membranes were post-treated with liquid nitrogen quick-freezing for several seconds. Finally, the purified and dried BC carriers were obtained after freeze-drying for 24 h. The freeze-drying temperature was fixed at −80 °C (cold trap temperature).

### 2.3. Preparation of TiO_2_/BC and TiO_2−x_/CBC Adsorbents

In a typical synthesis of TiO_2_/BC, 36 mL of deionized water was mixed with 4 mL of HCl, followed by adding different volumes of TBT (1, 2, 3, and 4 mL, separately). For the preparation of TiO_2_/BC samples, about 100 mg pure BC precursor was used after purification. Subsequently, the BC membranes were placed into a Teflon reactor and reacted at 90 °C for 9 h. The resultant solid materials were washed with ethanol and distilled water and then freeze-dried for 6 h to obtain TiO_2_/BC. According to the different amount of TBT, the intermediate products were denoted as TiO_2_/BC-1, TiO_2_/BC-2, TiO_2_/BC-3, and TiO_2_/BC-4, respectively.

**Figure 1:**
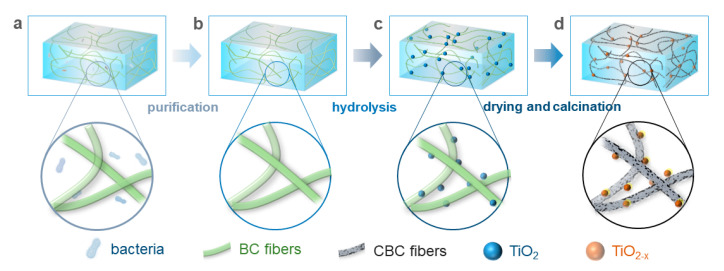
In situ synthesis of 3D structured TiO_2−x_/CBC containing four states of (**a**) natural BC membranes, (**b**) purified BC membranes, (**c**) TiO_2_/BC after hydrolysis, and (**d**) TiO_2−x_/CBC after drying and calcination.

The sample of TiO_2_/BC-4 was used for the study of the calcination temperature. In a typical synthesis of TiO_2−x_/CBC (Figure 1), TiO_2_/BC precursors were calcined under the same conditions, including a calcination atmosphere (flowing pure N_2_) and the same heating rate (5 °C/min) and calcination time (1 h). The only key variable of the calcination process was the calcination temperature (T*_c_*) of four different points (200 °C, 300 °C, 400 °C, and 500 °C). Correspondingly, these partially carbonized composites were labeled as TiO_2−x_/CBC-200, TiO_2−x_/CBC-300, TiO_2−x_/CBC-400, and TiO_2−x_/CBC-500.

### 2.4. Characterization

The phase and crystallinity information of the composites were qualitatively analyzed using X-ray diffraction (XRD, Rigaku Co., Ltd., Tokyo, Japan). The analysis employed Cu Kα radiation (λ = 1.54056 Å), with the instrument operating at 40 kV and 100 mA. A JSM-6360LA scanning electron microscope (SEM, JEOL, Tokyo, Japan) and a JEM-2100 transmission electron microscope (TEM, JEM-2100, JEOL, Tokyo, Japan) were used to observe the microscopic morphology and surface structure of all the composites. The samples were tested for nitrogen adsorption–desorption using a fully automated specific surface area analyzer (Micromeritics ASAP 2000, Norcross, GA, USA), and the pore size and specific surface area of the materials were obtained by analyzing the isothermal adsorption–desorption curves using the BET method. The thermal stability was analyzed via thermogravimetric analysis (TGA, TG209F3 Tarsus, Netzsch, Selb, Germany), and X-ray photoelectron spectroscopy (XPS, Thermo Scientific KAlpha, Waltham, MA, USA) was performed to characterize the compositions of materials. In addition, a Fourier transform infrared spectrometer (FTIR, Nicolet iS50, Madison, WI, USA) and Raman spectroscopy were used to analyze the surface functional groups and chemical compositions of the samples. Solid UV-*vis*-NIR diffuse reflectance spectroscopy (DRS, UV-3600i Plus UV-*vis*-NIR, Shimadzu, Kyoto, Japan) was used to characterize the band gap widths of the samples. To assess the surface charge density and the intensity of electrostatic interactions on the synthesized nanoparticles, the Zeta potential of the samples at different calcination temperatures was measured using a ZEN3600 Laser Particle Size and Zeta Potential Analyzer (Zetasizer Software 7.11).

### 2.5. Evaluation of the Adsorption Activity

Firstly, our study verified the single-component adsorption activities of as-prepared TiO_2−x_/CBC composites using MO, MG, RhB, TC, and MB as typical probes. The concentrations of all the above solutions were kept at 20 mg/L. To identify the separation effect, a mixed-dye solution (50 mL) was formed from MB and MO with equal concentrations of 20 mg/L. The quality of each adsorbent was fixed at 10 mg for each test. After adsorption for a period of time, 2 mL of the reaction suspension samples was collected and centrifuged (8000 rpm, 3 min) before the absorption spectra were measured. The absorbance values near 664 nm and 465 nm were analyzed using a UV-vis spectrophotometer (Shanghai Precision & Scientific Instrument Co., Ltd., Shanghai, China) to calculate the concentrations of MB and MO, respectively. The removal efficiency (*R*) of MB or MO by different samples is expressed as Equation (1) [42]:

(1) $$R=\frac{C_{0}-C_{t}}{C_{0}}\times 100\%$$

where C_0_ is the initial MB or MO concentration, and C_t_ is the MB or MO concentration after the reaction for time t. The separation efficiency for the mixture of organic dyes, which, in this context, represents selectivity, can be evaluated using Equation (2) [43]:

(2) $$\text{Separation efficiency }(\%)=\frac{{C(MO)}_{t}}{{C(MO)}_{t}+{C(MB)}_{t}}\times 100\%$$

where C(MO)_t_ *C(MO)_t_* and C(MB)_t_ *C(MB)_t_* are the concentrations of MO and MB dyes remaining in the solution after the adsorption.

The adsorption kinetics was studied using the pseudo-second-order model, as shown below [44]:

(3) $$\;t/Q_{t}=1/k_{2}{Q_{e}}^{2}+t/Q_{e}$$

In Equation (3), *k*_2_ is the pseudo-second-order rate constant (mg/g·min), *Q_e_* is the adsorption capacity at equilibrium, and *Q_t_* is the adsorption capacity at time *t*. The values of *Q_e_* and *k*_2_ can be obtained from the linear relationship plot of Q_t_ *Q_t_*/*t* versus *t.*

## 3. Results and Discussion

### 3.1. Material Characterization

**Figure 2:**
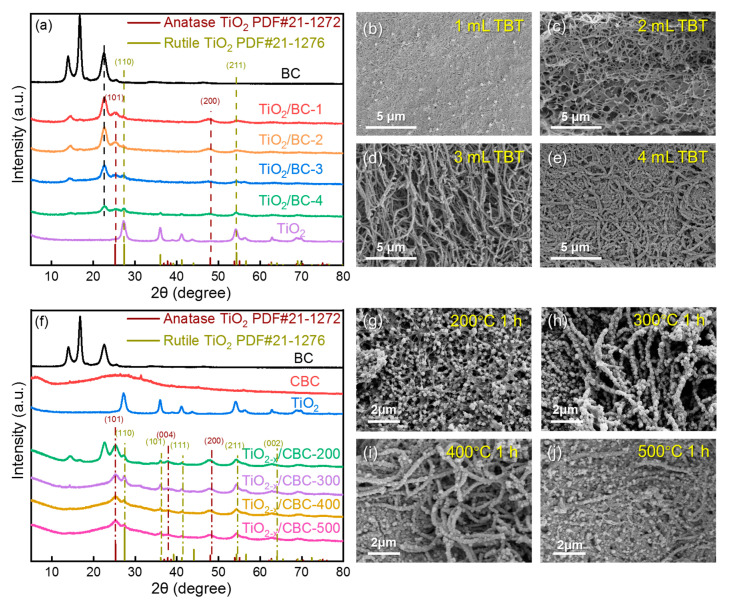
(**a**) XRD patterns and (**b**–**e**) SEM images of TiO_2_/BC with various TBT concentrations; (**f**) XRD patterns and (**g**–**j**) SEM images of TiO_2−x_/CBC under different calcination temperatures (T*_c_*).

Figure 2 shows the XRD patterns and SEM images of TiO_2_/BC with four different concentrations of TBT (Figure 2a–e), as well as four different calcination temperatures (T*_c_*) (Figure 2f–j). Two typical peaks at 14.5° and 22.8° corresponding to the (110) and (200) planes of cellulose clearly existed in both BC and TiO_2_/BC. The XRD pattern of pure TiO_2_ exhibited a mixture of anatase and rutile phases. Moreover, from the red line to the green line in Figure 2a, the (110) peak intensities of TiO_2_ varies with increasing amounts of TBT, indicating an increase loading of TiO_2_. The intensity ratio of (200) peaks at 22.8° among pure BC (black line), TiO_2_/BC-1 (red line), and TiO_2_/BC-4 (green line) was 100:78:20, respectively. The detailed peak intensities can be found in Table S1 and Figure S1. The variation in TiO_2_ peak intensity between TiO_2_/BC-3 and TiO_2_/BC-4 is minimal, indicating that the available sites on the fibers had been occupied, preventing further loading. As shown in Figure 2b–e, BC carriers showed a typical three-dimensional structure with plenty of empty spaces, which helped TBT molecules quickly diffuse and anchor onto BC fibers. Under the condition of 1 mL (Figure 2b) and 2 mL (Figure 2c) of TBT, only a few TiO_2_ precursors were loaded onto BC fibers. When the amount of TBT grew up to 3 mL (Figure 2d) and 4 mL (Figure 2e), the gaps between BC fibers were partially shortened, while the diameter fibers became larger due to the adhesion effect in hydrolysis. Figure 2f shows the XRD patterns of TiO_2−x_/CBC under different T*_c_* values. The characteristic peaks of CBC could be clearly seen in the samples of TiO_2−x_/CBC-300, TiO_2−x_/CBC-400, and TiO_2−x_/CBC-500. Interestingly, this kind of effect caused the lowest ratio of A/R (1.25) at 300 °C. But when using a high temperature of 500 °C, the ratio of A/R grew up to 2.50. The reason might be the interaction under a suitable T*_c_* inhibited the growth of crystalline structures as well as the transformation from anatase to rutile phase. In our study, the calcination temperature was selected between 300 °C and 500 °C, which was much lower than the effective transition temperature. As shown in Table S2, carbon-based networks under a higher temperature (e.g., 400 °C and 500 °C), which derived from BC, significantly inhibit the phase transition process from the anatase phase to the rutile phase of TiO_2_ [45,46]. For comparison, Figure 2g–j present the morphologies of TiO_2−x_/CBC under different T*_c_* values. Obviously, when the T*_c_* was 200 °C (Figure 2g) or 300 °C (Figure 2h), ultrasmall TiO_2−x_ particles were well distributed on the surface of the CBC fibers. However, as the T*_c_* increased to 400 °C, partial aggregation of TiO_2−x_ appeared in Figure 2i. Moreover, the condition of 500 °C caused serious aggregation of and structure damage to the CBC fibers (Figure 2j). The element mapping spectra and EDS energy spectrum of TiO_2−x_/CBC-300 can be seen in Figure S2. The atomic ratio of the C, Ti, and O elements, as determined via EDS, is presented in Table S3.

**Figure 3:**
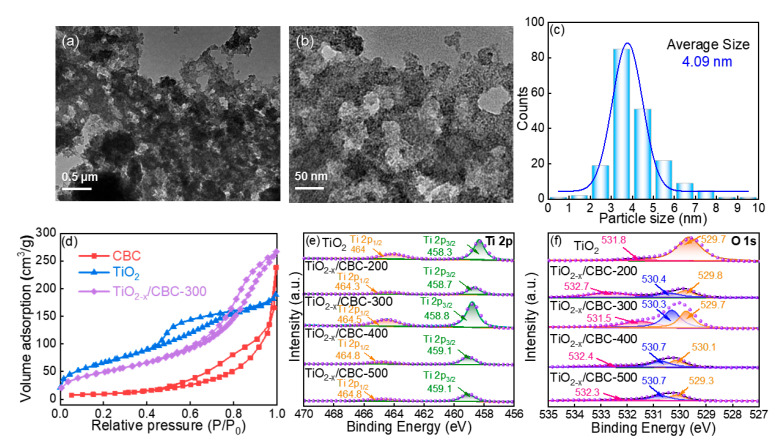
(**a**,**b**) TEM images of TiO_2−x_/CBC-300; (**c**) size distribution of TiO_2−x_ nanoparticles; (**d**) N_2_ adsorption–desorption isotherms of TiO_2−x_/CBC-300; XPS spectra of (**e**) Ti 2p and (**f**) O 1s of TiO_2−x_/CBC-300 (dot line represents the original data).

The detailed morphologies of metallic oxides on CBC fibers were analyzed via TEM (Figure 3a,b). Firstly, many “windows” with a small size (<50 nm) were observed between fibers, which were smaller than blank CBC (Figure S3). In addition, a large number of small particles were loaded on the surface of CBC. These ultrasmall particles occupied the initial windows within CBC membranes, resulting in a certain decrease in the specific surface area of TiO_2−x_/CBC-300. It is also demonstrated in subsequent sections through N_2_ adsorption–desorption isotherms. After measuring over 150 particles (Figure S4), the particle size distribution was determined, as shown in Figure 3c. The average size of TiO_2−x_ was confirmed to be ~4.09 nm, which proved that the TiO_2−x_ particles were well dispersed, with an ultrasmall particle size. To confirm the pore size and surface areas of adsorbents, the N_2_ adsorption–desorption isotherms of blank CBC, pure TiO_2_, and TiO_2−x_/CBC-300 and the relative results are shown in Figure 3d and Table S4. The adsorption–desorption hysteresis loop of TiO_2_ was relatively narrow, and the BET surface area (S_BET_) for pure TiO_2_ was 34.7 m^2^/g. In contrast, the desorption curve of CBC fibers became flatter with increasing pressure, suggesting that the pores of CBC were primarily composed of crack-like micropores. The S_BET_ of CBC was 245.6 m^2^/g, much larger than BC fibers (77.82 m^2^/g) [47]. The adsorption isotherm of TiO_2−x_/CBC-300 exhibited a type IV isotherm with a high nitrogen adsorption rate at low pressures and a large hysteresis loop, indicating the presence of a large number of mesoporous structures. Compared with CBC, the S_BET_ of TiO_2−x_/CBC-300 decreased to 182.6 m^2^/g due to the possibility that ultrasmall TiO_2−x_ particles could easily fill the cracks and defects of CBC fibers. Considering the much smaller molecular sizes of the four organic dyes used in this work than the pore size of TiO_2−x_/CBC (Table S4), the pore size effect can be excluded.

XPS analysis was performed to investigate the surface chemistry and valence states (Figure 3e–f, Figures S5 and S6). Compared with pure TiO_2_, all the binding energies of the Ti 2p_1/2_ and Ti 2p_3/2_ peaks increased after combining CBC with TiO_2−x_ (Figure 3e). Moreover, a higher calcination temperature induced the shift to higher binding energy, which indicated that a part of the Ti-O-Ti bonds were substituted by Ti-O-C and O=C-O-Ti bonds due to the higher electronegativity of the carbon element. As shown in Figure 3f, the binding energies of 529.3~530.1 eV, 530.3~530.7 eV, and 531.5~532.7 eV correspond to lattice oxygen (Ti-O), adsorbed oxygen on oxygen vacancies, and surface hydroxyl oxygen, respectively. As we know, oxygen vacancy (V*_o_*) can directly determine the performance of selective adsorption [48,49]. It is worth to mention that the T*_c_* of 300 °C led to the highest concentration of oxygen vacancy (V*_o_*). Furthermore, a weak signal around the binding energy of 531.5 eV in TiO_2−x_/CBC-300 exhibited some kind of hydroxyl oxygen residue under the temperature of 300 °C. The electron paramagnetic resonance (EPR) spectra of all samples are shown in Figure S7. The unpaired electrons associated with the defects are identified by a single Lorentzian line at g = 2.003. The signal intensity of the EPR spectra corresponds to the concentration of oxygen vacancies. Based on the EPR, it can be inferred that the oxygen vacancy concentration is highest in TiO_2−x_/CBC-300, and this is consistent with the changes observed in the XPS. It means that either a too high or too low T*_c_* could greatly influence the interaction between TiO_2_ and biomass fibers and further limit the oxygen vacancy concentration.

**Figure 4:**
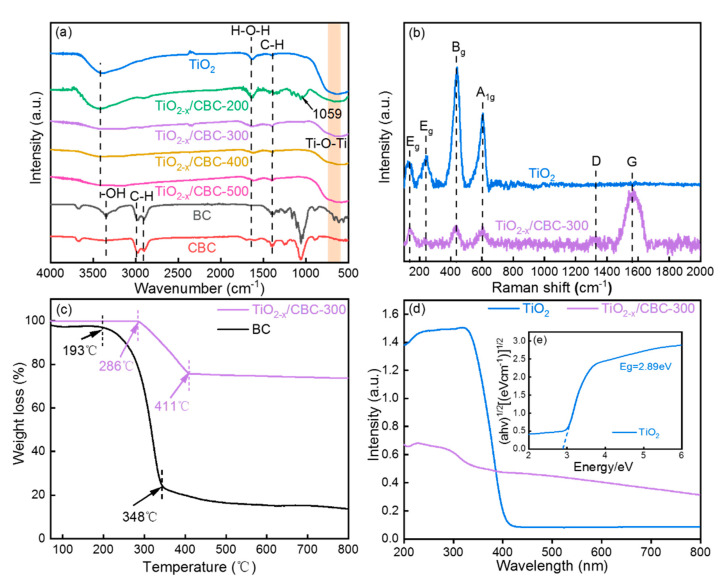
(**a**) FT-IR spectra of TiO_2_, BC, CBC, and TiO_2−x_/CBC under different calcination temperatures; (**b**) Raman spectra of TiO_2_, and TiO_2−x_/CBC-300; (**c**) TG profiles of BC and TiO_2−x_/CBC-300; (**d**) UV-vis DRS spectra and (**e**) Kubelka–Munk plots of TiO_2_ and TiO_2−x_/CBC-300.

FTIR spectra were employed to analyze the surface functional groups on the pure TiO_2_, blank BC, CBC, and TiO_2−x_/CBC scaffolds, respectively. In Figure 4a, peaks of BC (black line) and CBC (red line) around 1059 cm^−1^, 2950 cm^−1^, and 3349 cm^−1^ were caused by symmetric stretching of C-O, deformation vibrations of the C-H bond, and stretching vibrations of O-H groups, respectively. For comparison, from TiO_2−x_/CBC-200 (green line) to TiO_2−x_/CBC-500 (pink line), the peak intensities of the C-O, C-H, and H-O-H groups were weakened, while the characteristic peak of TiO_2_, such as Ti-O-Ti, was gradually enhanced. The FTIR results indicate a strong interaction between titanium oxides and the CBC carriers. Furthermore, the Raman spectra of TiO_2_ and TiO_2−x_/CBC-300 are shown in Figure 4b. Compared with pure TiO_2_, the TiO_2−x_/CBC-300 had two extra peaks that belonged to the D band (1334 cm^−1^) and G band (1585 cm^−1^) of CBC. The value of I_d_/I_g_ is only 0.24, which indicated few defects and a high degree of graphitization. Additionally, the E_g_ mode of anatase TiO_2_ was observed at 141.4 cm^−1^ and the B_g_, A_1g_, and E_g_ modes of rutile TiO_2_ were confirmed at 438.9 cm^−1^, 605.8 cm^−1^, and 237 cm^−1^, respectively. The results were consistent with the XRD analysis that both the anatase phase and rutile phase were formed. In Figure 4c, TG analysis shows the thermal stability of TiO_2−x_/CBC-300. The decomposition temperature range moved from 193 °C–348 °C to 286 °C–411 °C. We chose 300 °C as the target temperature. At this point, the BC is in a state between initial and complete decomposition. This moderate decomposition generates small molecular fragments and new active sites, which are crucial for anchoring and evenly dispersing TiO_2−x_ nanoparticles, thus enhancing the material’s performance. The TG results confirmed that the anchoring of TiO_2_ enhanced the thermal stability of natural BC fibers. As presented in Figure 4d, the UV-vis light absorption properties of the TiO_2_ and TiO_2−x_/CBC-300 were studied. The band-gap energy of the TiO_2_ was determined to be 2.89 eV by using Kubelka–Munk models (Figure 4e). Compared with pure TiO_2_, the band-gap energy of the TiO_2−x_/CBC-300 significantly reduced and shifted towards the visible light region (red-shift), which could lead to a better optical response. In Figure S9, the highest photocurrent response achieved by TiO_2−x_/CBC-300 illustrates the best separation capability of electron–hole pairs. PL spectrum observation also shows that TiO_2−x_/CBC-300 has a much lower PL intensity (Figure S9) than that of others. That is to say, TiO_2−x_/CBC-300 obtains not only the optimal light absorption capacity but also excellent performance in electron–hole separation. The photocatalytic degradation effect of MB by TiO_2−x_/CBC adsorbents is listed in Figure S10. As shown in Figure S10, during the 180 min photocatalytic degradation process, the degradation capabilities of the various catalysts towards MB exhibited significant differences. Specifically, the TiO_2−x_/CBC-300 catalyst demonstrated the highest degradation efficiency, followed by TiO_2−x_/CBC-400 and TiO_2−x_/CBC-500, while pure TiO_2_ and TiO_2−x_/CBC-200 exhibited relatively weaker degradation capabilities. This result not only validates the photocatalytic activities of these composites but also provides a potential application value for regeneration via photocatalytic degradation.

### 3.2. Adsorption Performance

**Figure 5:**
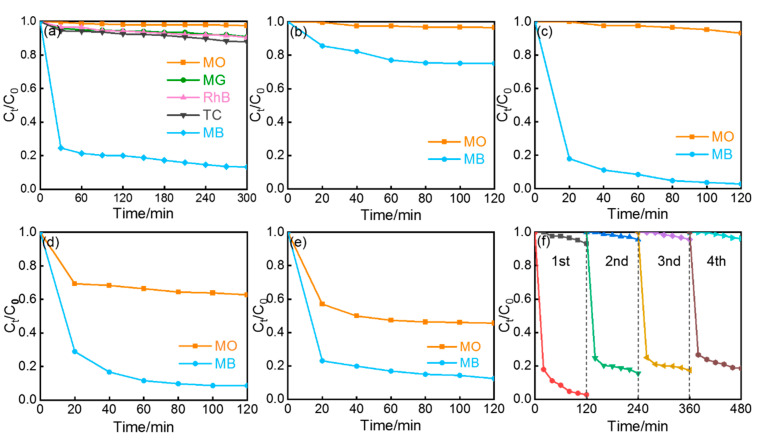
(**a**) Single-component adsorption activities of TiO_2−x_/CBC-300 for five pollutants. (**b**) Double-component selective adsorption activities of (**b**) TiO_2−x_/CBC-200, (**c**) TiO_2−x_/CBC-300, (**d**) TiO_2−x_/CBC-400, and (**e**) TiO_2−x_/CBC-500. (**f**) Cycling test of TiO_2−x_/CBC-300 in the dark.

**Figure 6:**
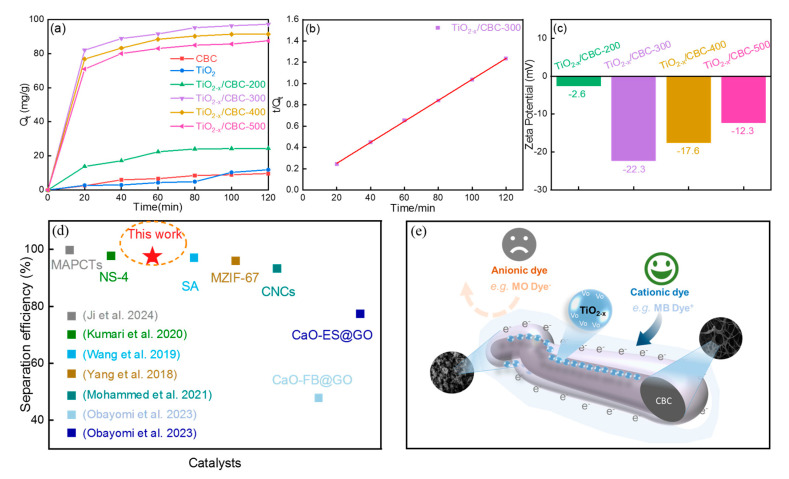
(**a**) Adsorption kinetics of blank CBC, pure TiO_2_, and TiO_2−x_/CBC under different calcination temperatures, (**b**) pseudo-second-order model of TiO_2−x_/CBC-300, (**c**) Zeta potentials of TiO_2−x_/CBC adsorbents under four different calcination temperatures, (**d**) the separation efficiencies for MB/MO in the reported adsorbents (MAPCTs [24], NS-4 [43], SA [50], MZIF-67 [51], CNCs [52], CaO-ES@GO and CaO-FB@GO [53]), and (**e**) possible mechanism for the high selectivity of TiO_2−x_/CBC-300.

To investigate the adsorption performance of TiO_2−x_/CBC for organic dyes, we analyzed the adsorption curves for both individual and mixed-dye solutions. Figure 5a compared the single-component adsorption activities for five different organic pollutants, including MO, MG, RhB, TC, and MB, separately (Figure S11). Taking TiO_2−x_/CBC-300 as an example, MB exhibited the highest adsorption effect while achieving a removal efficiency (R) of 86.74% after 300 min. In contrast, the R values for MO, MG, RhB, and TC were relatively lower at 2.45%, 9.11%, 9.42%, and 12.16%, respectively. MB and MO, as two representative organic dye compounds, have been extensively utilized in various industrial applications and scientific research fields. Further analysis of mixed solutions of MO/MB revealed distinct adsorption characteristics under different calcination temperatures. When the T*_c_* condition was 200 °C, the weak removal rate for both MB and MO stayed at a very low level (Figure 5b). Compared with CBC and pure TiO_2_ (Figure S12), however, TiO_2−x_/CBC-300 showed significantly enhanced selectivity for the mixed solution after 120 min, with an ultrahigh *C_t_/C_0_* value for MO (97.2%) and an ultralow value for MB (6.9%) in Figure 5c. Moreover, the selective adsorption performance of TiO_2−x_/CBC-400 and TiO_2−x_/CBC-500 gradually decreased with higher calcination temperatures of 400 °C (Figure 5d) and 500 °C (Figure 5e). Overall, when the calcination temperature (T*_c_*) varied from 200 °C to 500 °C, the separation efficiencies of the TiO_2−x_/CBC adsorbents for the mixed dyes at 120 min were 56.27%, 97.07%, 87.97%, and 78.56%, respectively. As a result, TiO_2−x_/CBC-300 had a superior selective adsorption ability due to its 3D cross-linked structure equipped with oxygen vacancy-rich surfaces. Notably, it was found consistent with the conclusion in terms of their surface Zeta potentials, as shown in Figure 6c. Therefore, the excellent selective adsorption capacity of TiO_2−x_/CBC-300 towards cationic organic dyes can be attributed to the increased surface negative charge stemming from the synergism between vacancy-rich TiO_2_ and π–π interaction. As we know, the stability of adsorbent is critical for practical applications. Thus, the cycling performance of TiO_2−x_/CBC-300 was carefully studied in the mixed solution of MB and MO. The solids were collected after adsorption and washed three times with water and methanol alternately under the condition of ultrasonication for 5 min in a 10 mL centrifuge tube. After being gathered via centrifugation, the adsorbent was then dried in an oven at 60 °C. After 6 h, the composite was ready for reuse in the next cycle. After four cycles of the adsorption–desorption process under dark conditions, the value of the separation efficiency remained at 83.84%, representing a slightly decrease by only 13.23% compared to the initial value of separation efficiency (Figure 5f). The SEM (Figure S13) patterns of TiO_2−x_/CBC-300 after regeneration exhibited similarity to those before the reaction, with no significant differences observed. We confirmed that the system of TiO_2−x_/CBC has both excellent selective adsorption and good stability for separating mixed dyes.

Figure 6a shows the adsorption kinetic curves of MB on CBC, TiO_2_, and TiO_2−x_/CBC. Based on the pseudo-second-order fitting plots for MB, within the first 20 min, four TiO_2−x_/CBC samples reached over 70% of the final saturated adsorption capacity, which indicated the quick adsorption abilities of TiO_2−x_/CBC. The final saturated adsorption capacities of blank CBC, pure TiO_2_, TiO_2−x_/CBC-200, TiO_2−x_/CBC-300, TiO_2−x_/CBC-400, and TiO_2−x_/CBC-500 after 120 min adsorption were 9.6, 11.9, 24.4, 97.2, 91.4, and 87.5 mg/g. In Figure 6b and Table S5, it can be seen that the correlation coefficient (R^2^ = 0.998) from the pseudo-second-order model is was higher than that from the pseudo-first-order model (R^2^ = 0.878), indicating that this TiO_2−x_/CBC system was controlled by liquid film diffusion, as well as internal diffusion and chemical adsorption. Moreover, the Zeta potential of composite is also crucial, particularly for the separation of anionic and cationic dyes. Figure 6c presents the Zeta potential profiles for TiO_2−x_/CBC across different calcination temperatures. TiO_2−x_/CBC-200 showed a weak Zeta potential of −2.6 mV, indicating weak electrostatic attraction for cationic dyes, correlating with the poor dye adsorption capacity shown in Figure 5b. In contrast, TiO_2−x_/CBC-300 achieved the strongest Zeta potential of −22.3 mV, while higher calcination temperatures of 400 °C and 500 °C yielded the Zeta potentials of −17.6 mV and −12.3 mV, respectively. Electrostatic interaction and π–π interaction were determined as the main driving forces, and the 3D cross-linked structure also contributed to the adsorption process. In conclusion, there is a strong dependency relationship among the (i) V*_o_* concentration, (ii) surface electronegativity, and (iii) separation efficiencies. Compared with related publications (Figure 6d, Table S6), our TiO_2_-BC based material is convenient for synthesis with a higher separation efficiency. A possible mechanism for the high selectivity of TiO_2−x_/CBC-300 is shown in Figure 6e. From a preparation perspective, the carbonization temperature (T*_c_*) plays a key role in adjusting the selective adsorption effects of anionic/cationic dyes.

## 4. Conclusions

In this work, we proposed a novel route for the in situ fabrication of ultrasmall TiO_2−x_ particles on CBC fibers using a facile hydrolysis–calcination process. The rich -OH groups of BC ensured a well-dispersed and size-controlled synthesis of TiO_2_ particles. Additionally, the optimized calcination temperature of 300 °C introduced the highest oxygen vacancy and the biggest Zeta potential (absolute value) to the 3D scaffold materials. Regarding the investigation of adsorption performance, our initial efforts were concentrated on single-dye adsorption experiments, involving MB, MO, MG, RhB, and TC. The TiO_2−x_/CBC material manifested a robust adsorption capacity of 101.4 mg/g for MB. This value was markedly higher compared to the adsorption capacities for the other single dyes examined in this study. Subsequently, a binary mixture of MB and MO was prepared and subjected to adsorption tests. TiO_2−x_/CBC-300 exhibited an outstanding adsorption performance, especially high selectivity for mixed pollutants. As for the mixture of MB/MO, the separation efficiency of TiO_2−x_/CBC-300 was 97.07%. Moreover, the adsorbents could be easily regenerated following methanol washing. After four cycles of adsorption–desorption in a dark atmosphere, the separation efficiencies of the TiO_2−x_/CBC-300 remained over 83.84% for the target dye. This work provides compelling evidence for the huge potential of TiO_2_-based materials in designing high-efficiency adsorbents with excellent selectivities.

## Data Availability

The original contributions presented in this study are included in the article/Supplementary Material. Further inquiries can be directed to the corresponding authors.

## Supplementary Materials

The supporting information can be downloaded at: https://www.mdpi.com/article/10.3390/nano15070514/s1. See Refs [24,43,50,51,52,53,54,55,56,57].
