# Supplementary material for: In Situ Anchored, Ultrasmall, Oxygen Vacancy-Rich TiO2−x on Carbonized Bacterial Cellulose for the Efficient Adsorption and Separation of Organic Pollutants
Source: Nanomaterials (Basel). 2025 Mar 28;15(7):514. doi: 10.3390/nano15070514 (PMC11990132; doi:10.3390/nano15070514)
Supplement: Supplementary file 1 [file nanomaterials-15-00514-s001.zip › nanomaterials-3509091-supplementary.pdf]

Supplementary materials

In-situ **anchored ultrasmall, oxygen vacancy-rich TiO_{2-x} on carbonized bacterial cellulose for efficient adsorption and separation of organic pollutants**

Man Zhou[a*], Yanli Zhou[a], Minmin Ni[a], Yuzhe Zhang[a], Song Xu[a], Hao Ma[b], Jian Zhou[c], Jin Zhao[d], Liwei Lin[a] and Zhongyu Li[a*]

[a] Jiangsu Province Key Laboratory of Fine Petrochemical Engineering, Changzhou University, Changzhou 213164, China

[b] BGRIMM Technology Group, Daxing, Beijing, 102600, China.

[c] Zhongxi Rare Earth New Materials Co., Ltd, Changzhou, 213164, China.

[d] School of Materials Science and Engineering, Nanjing University of Posts and Telecommunications, Nanjing, 210023, China

[*] Corresponding author: Man Zhou, zhouman@cczu.edu.cn

Zhongyu Li, zhongyuli@mail.tsinghua.edu.cn

Tel.: +86-519-86330088; Fax: +86-519-86330088

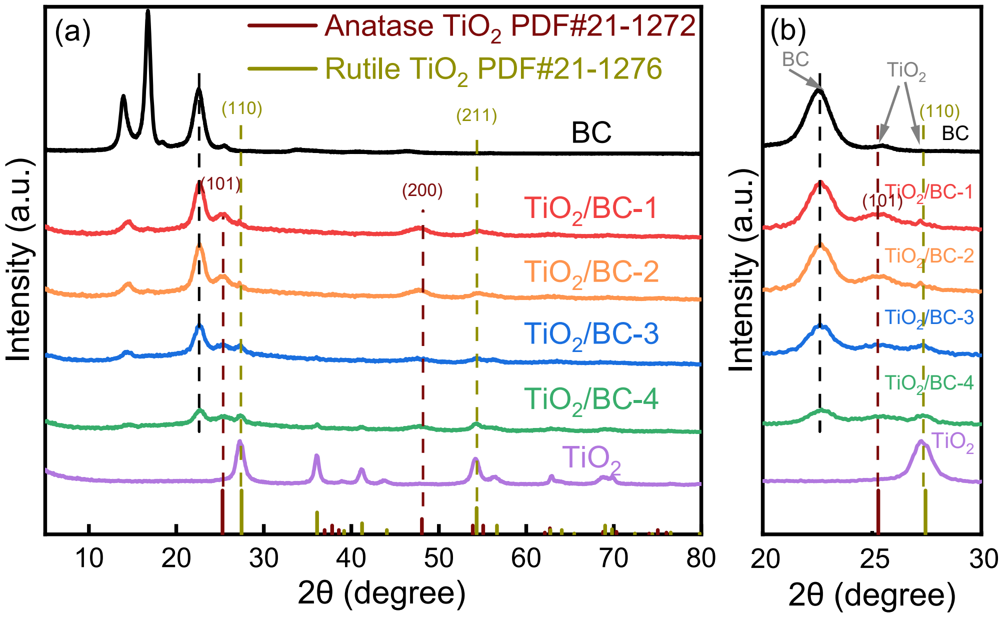

Figure S1 (a) XRD patterns and (b) enlarged curves between 20° and 30° of TiO_2/BC with various TBT concentrations.

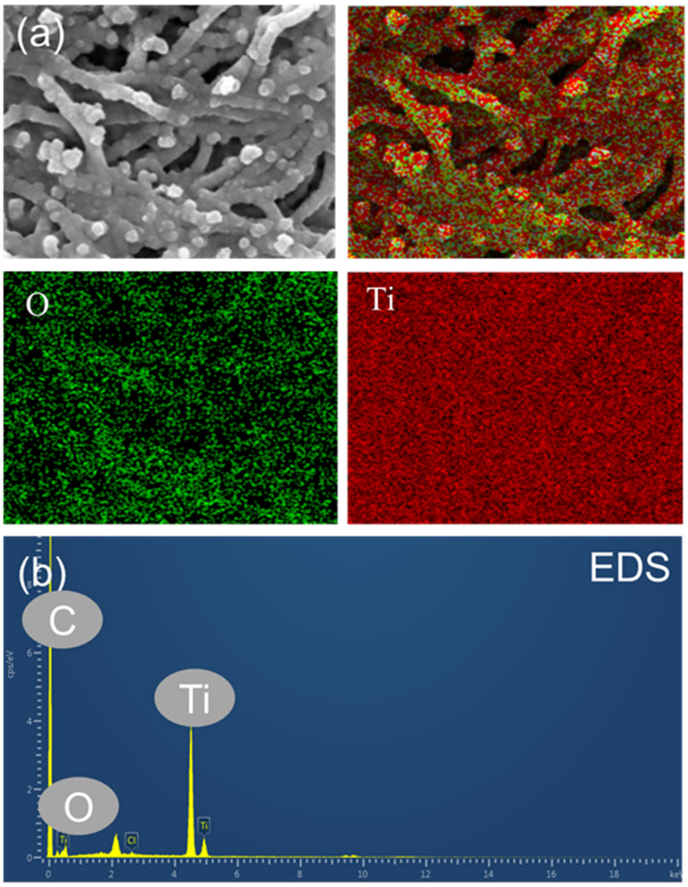

Figure S2. (a) Ti (red), O (green) element mapping spectra of TiO_{2-x}/CBC-300, (b) EDS energy spectrum analysis of TiO_{2-x}/CBC-300.

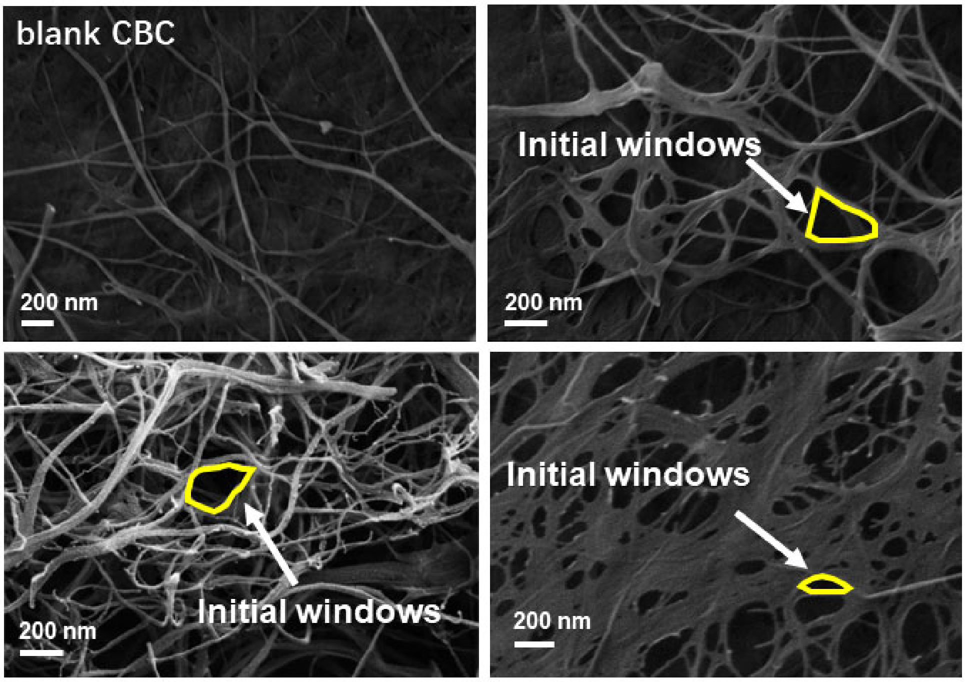

Figure S3. Typical SEM images of blank CBC scaffold.

(yellow ring refers to the initial windows in CBC fibers)

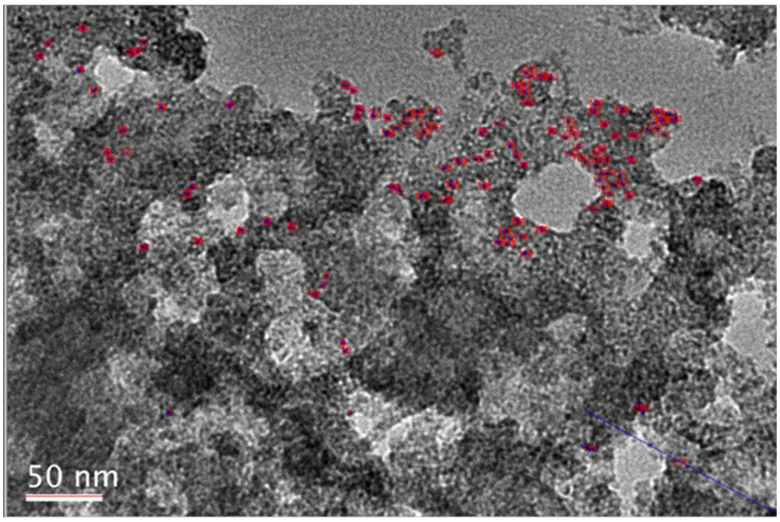

Figure S4. The original TEM image of TiO_{2-x}/CBC-300 measured by software Nano Measurer 1.2.

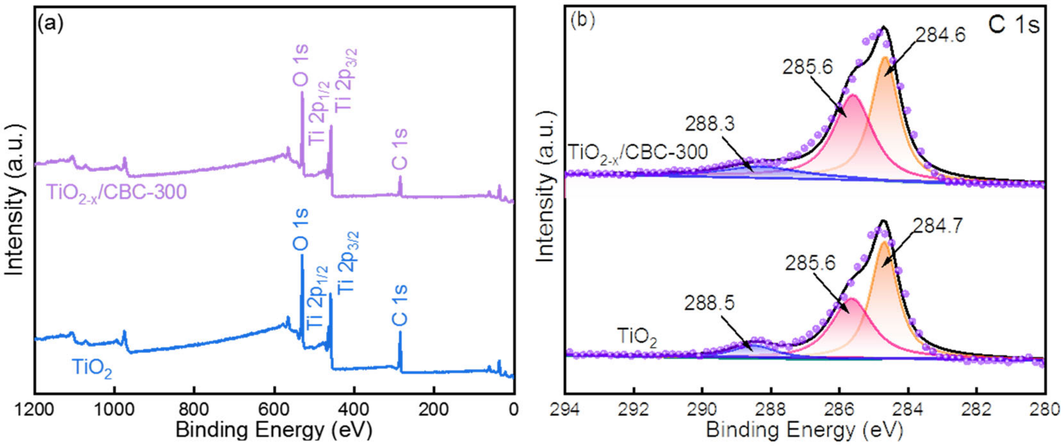

Figure S5. (a) Survey XPS spectra of TiO_2 and TiO_{2-x}/CBC-300, (b) high resolution XPS spectra of

C 1s in TiO_2 and TiO_{2-x}/CBC-300.

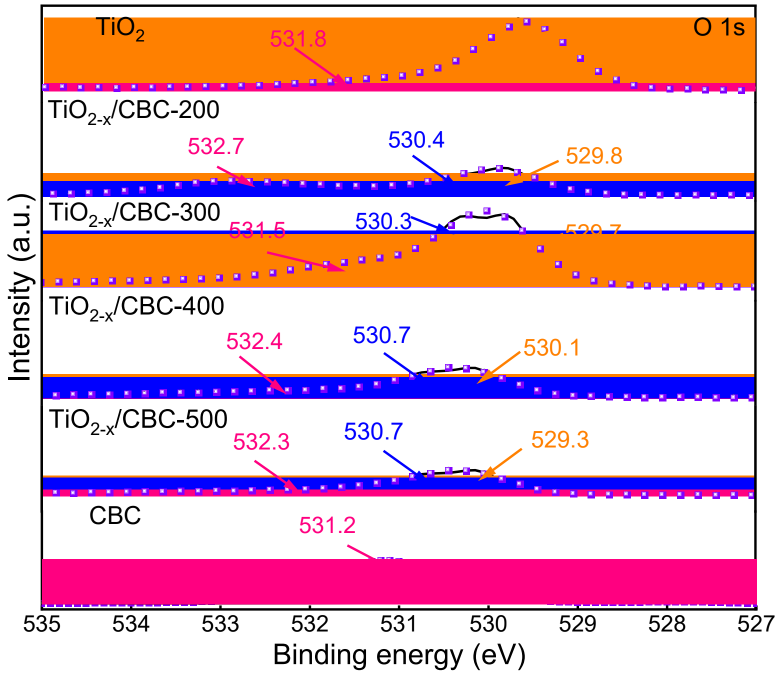

Figure S6. XPS spectra of O 1s of TiO_{2-x}/CBC-300.

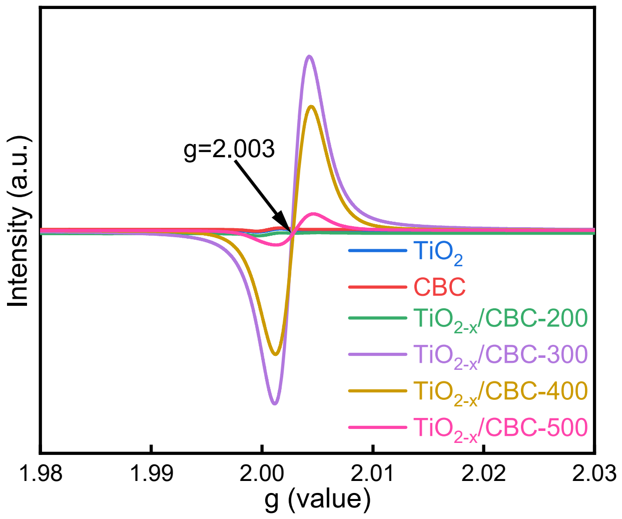

Figure S7. The EPR spectra of pure TiO_2 and TiO_{2-x}/CBC under different calcination temperatures (Tc).

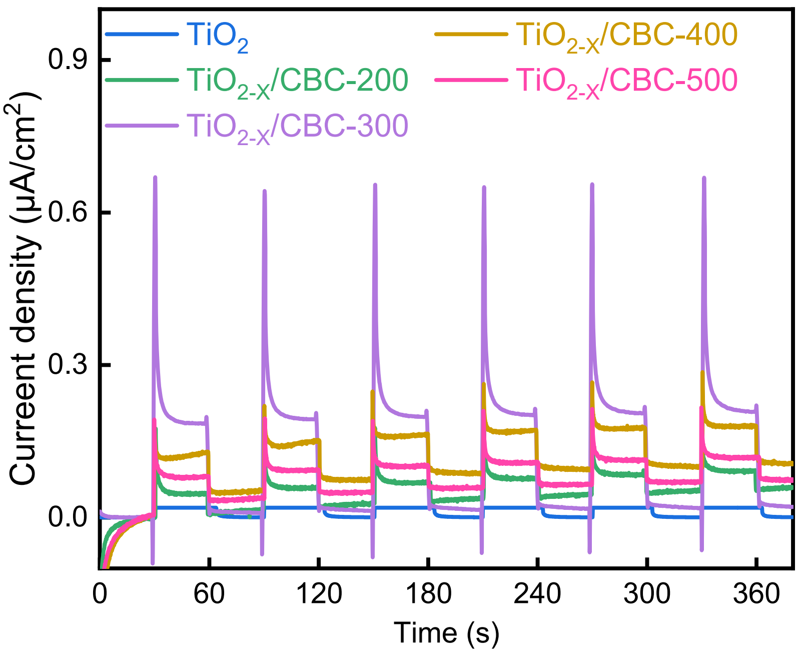

Figure S8. The photocurrent spectra of pure TiO_2 and TiO_{2-x}/CBC under different calcination temperatures (Tc).

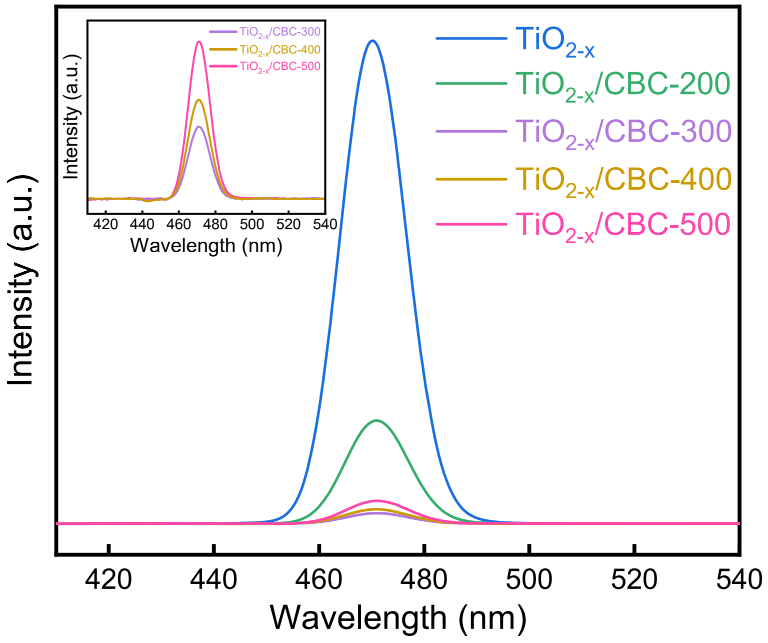

Figure S9. The PL spectra of pure TiO_2 and TiO_{2-x}/CBC under different calcination temperatures(Tc).

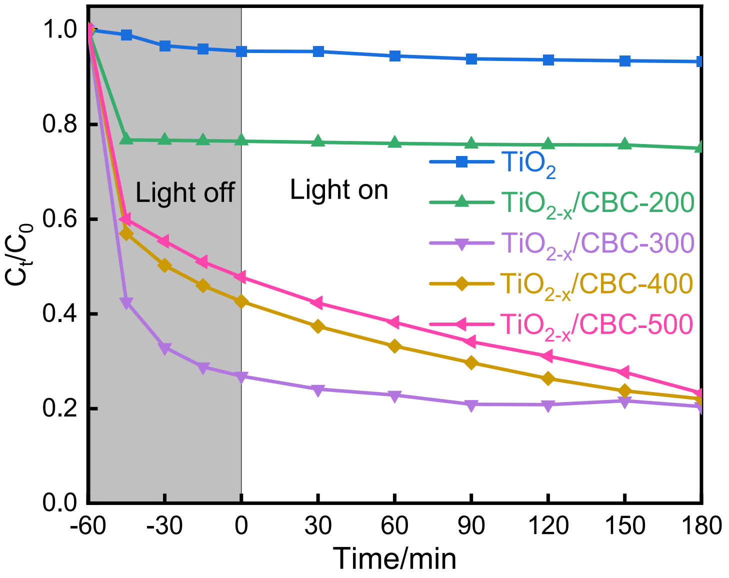

Figure S10. The photocatalytic degradation curves of pure TiO_2 and TiO_{2-x}/CBC under different calcination temperatures (T_c).

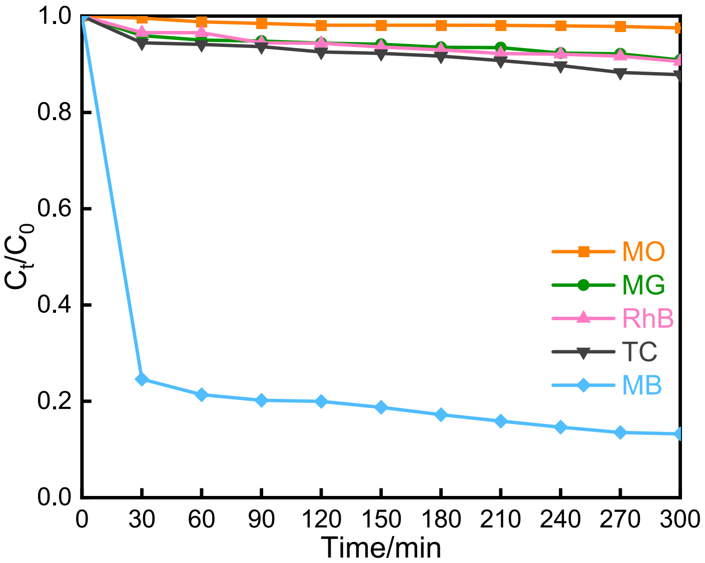

Figure S11. Adsorption performance diagram of TiO_{2-x}/CBC for different dyes and drugs

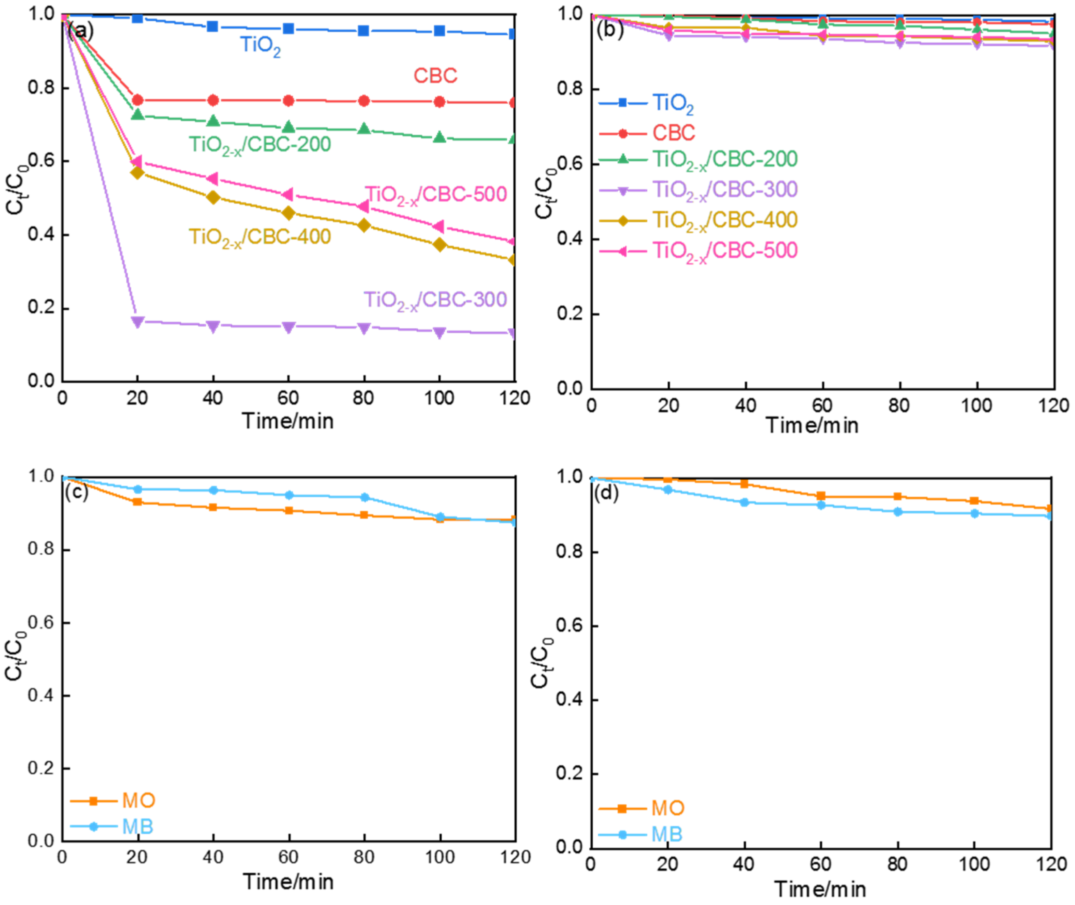

Figure S12. Adsorption performances of six adsorbents for removing single-component dye of (a) MB and (b) MO; Selective adsorption performances of (c) CBC, (d) TiO_2 in mixed MB/MO solution.

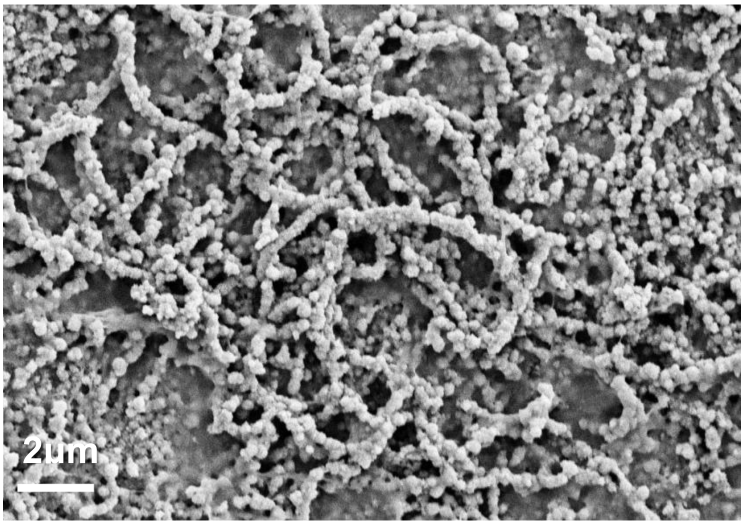

Figure S13. SEM images of TiO_{2-x}/CBC-300 after cyclic reactions

Table S1. The proportion of TiO_2 and BC in TiO_2/BC with different TBT concentrations.

Samples	Peak intensities of (200) plane at 22.8° (a.u.)	$I_{(sample)}/I_{(BC)}$
BC	6519.52	1
TiO_2/BC-1	5093.38	0.78
TiO_2/BC-2	4621.73	0.71
TiO_2/BC-3	3106.96	0.48
TiO_2/BC-4	1273.34	0.20

Table S2 The A/R values of composites subjected to various calcination temperatures.

Samples	A/R
TiO_2/CBC-200	2.60
TiO_2/CBC-300	1.25
TiO_2/CBC-400	2.47
TiO_2/CBC-500	2.5

Table S3 The atomic ratio of C, Ti and O elements calculated from EDS.

Element of TiO_2/CBC-300	Atomic percentage (%)
C	13.08
O	60.00
Ti	26.92
Total	100.00

Table S4. The values of S_{BET}, pore volume and pore diameter of TiO_2, CBC and TiO_{2-x}/CBC-300.

Sample	S_{BET} ($m^2 \cdot g^{-1}$)	Pore volume ($cm^3 \cdot g^{-1}$)	Pore diameters (nm)
CBC	245.59	0.40	34.49
TiO_2	34.68	0.32	4.71
TiO_{2-x}/CBC-300	182.66	0.45	8.92

Table S5. The equilibrium capacities, pseudo-second order rate constant and correlation coefficient R^2 of TiO_{2-x}/CBC at different calcination temperatures adsorbing MB.

Sample	Q_e (mg/g)	k_2 mg/(g·min)	R^2
TiO_{2-x}/CBC-200	30	0.00139	0.9997
TiO_{2-x}/CBC-300	101.4	0.00181	0.9997
TiO_{2-x}/CBC-400	95.7	0.00201	0.9998
TiO_{2-x}/CBC-500	91.2	0.00188	0.9998

Table S6. The separation efficiency of different catalysts for mixed dyes containing MB.

Name of adsorbents	Based materials	Separation efficiency	References
MAPCTs	porous carbon microtubes	99.70%	Ji et al. *Sep. Purif. Technol.* 2024 [1]
NS-4	nickel sulfide nanomaterials	97.75%	Kumari et al. *Colloid. Surface. A.* 2020 [2]
SA	sodium alginate	97.00%	Wang et al. *Carbohyd. Polym.* 2019 [3]
MZIF-67	magnetic ZIF-67	96.00%	Yang et al. *Chem. Eng. J.* 2018 [4]
CNCS	cellulose nanocrystals	93.27%	Mohammed et al. *Chem. Eng. J.* 2021 [5]
CaO-ES@GO	graphene oxide	77.34%	Obayomi et al. *Process Saf. Environ.* 2024 [6]
CaO-FB@GO	graphene oxide	47.81%	Obayomi et al. *Process Saf. Environ.* 2024 [6]
**TiO_{2-x}/BC**	**bacteria cellulose**	**97.07%**	**This work**
TiO_2/BTMSPA	organosilane	/	Tu et al. *Sep. Purif. Technol.* 2023 [7]
TiO_2-PVA	poly vinyl alcohol	/	Jaseela et al. *J. Mol. Liq.* 2019 [8]
TiO_2/MAC	macadamia	/	Thuy et al. *Mater. Res. Express* 2023 [9]
CF-PVA/GA@ APT/TiO_2	copper foam	/	Zeng et al *Sep. Purif. Technol.* 2025 [10]

1. L. Ji, L. Luo, D. Jin, X. Qin, Preparation of aligned porous carbon microtubes by a reactant permeation template method and the highly selective adsorption of methylene blue dye from wastewater, Sep. Purif. Technol. 332 (2024) 125884 https://doi.org/10.1016/j.seppur.2023.125884.

2. S. Kumari, A.A. Khan, A. Chowdhury, A.K. Bhakta, Z. Mekhalif, S. Hussain, Efficient and highly selective adsorption of cationic dyes and removal of ciprofloxacin antibiotic by surface modified nickel sulfide nanomaterials: kinetics, isotherm and adsorption mechanism, Colloid. Surface. A. 586 (2020) 124264 https://doi.org/10.1016/j.colsurfa.2019.124264.

3. Q. Wang, J. Ju, Y. Tan, L. Hao, Y. Ma, Y. Wu, H. Zhang, Y. Xia, K. Sui, Controlled synthesis of sodium alginate electrospun nanofiber membranes for multi-occasion adsorption and separation of methylene blue, Carbohyd. Polym. 205 (2019) 125-134 https://doi.org/10.1016/j.carbpol.2018.10.023.

4. Q. Yang, S. Ren, Q. Zhao, R. Lu, C. Hang, Z. Chen, H. Zheng, Selective separation of methyl orange from water using magnetic ZIF-67 composites, Chem. Eng. J. 333 (2018) 49-57

https://doi.org/10.1016/j.cej.2017.09.099.

5. N. Mohammed, H. Lian, M.S. Islam, M. Strong, Z. Shi, R.M. Berry, H. Yu, K.C. Tam, Selective adsorption and separation of organic dyes using functionalized cellulose nanocrystals, Chem. Eng. J. 417 (2021) 129237 https://doi.org/10.1016/j.cej.2021.129237.

6. K.S. Obayomi, S.Y. Lau, M.K. Danquah, J. Zhang, T. Chiong, O.V. Obayomi, L. Meunier, M.M. Rahman, A response surface methodology approach for the removal of methylene blue dye from wastewater using sustainable and cost-effective adsorbent, Process Saf. Environ. 184 (2024) 129-150 https://doi.org/10.1016/j.psep.2024.01.106.

7. M. Tu, J. Yu, J. Wang, X. Shi, Z. Fu, S. Hu, M. Zhong, Z. Fei, Coral-like TiO_2/organosilane hybrid particles with rapid adsorption of methyl orange, Sep. Purif. Technol. 309 (2023) 123000 https://doi.org/10.1016/j.seppur.2022.123000.

8. P.K. Jaseela, J. Garvasis, A. Joseph, Selective adsorption of methylene blue (MB) dye from aqueous mixture of MB and methyl orange (MO) using mesoporous titania (TiO_2) – poly vinyl alcohol (PVA) nanocomposite, J. Mol. Liq. 286 (2019) 110908 https://doi.org/10.1016/j.molliq.2019.110908.

9. L.T.T. Thuy, P. Thi Thuy, P. Thi Giang Anh, N.A. Tien, N. Thi Hong Bich, D.Q. Khieu, Simultaneous adsorption of malachite green, methyl orange, and rhodamine B with TiO_2/macadamia nutshells-derived activated carbon composite, Mater. Res. Express 10 (2023) 125602 https://doi.org/10.1088/2053-1591/ad16aa.

10. X. Zeng, M. Lu, Z. Xu, Z. Lin, F. Fan, Y. Xia, W. Li, M. Zhang, T. Chen, C. Zhou, Facile fabrication of superhydrophilic copper foam for rapid and efficient filtration of cationic dyes from water, Sep. Purif. Technol. 354 (2025) 128943 https://doi.org/10.1016/j.seppur.2024.128943.
